# A Dual-Band Flexible MIMO Array Antenna for Sub-6 GHz 5G Communications

**DOI:** 10.3390/s25113557

**Published:** 2025-06-05

**Authors:** Deepthi Mariam John, Tanweer Ali, Shweta Vincent, Sameena Pathan, Jaume Anguera, Bal Virdee, Rajiv Mohan David, Krishnamurthy Nayak, Sudheesh Puthenveettil Gopi

**Affiliations:** 1 Department of Electronics and Communication Engineering, Manipal Institute of Technology, Manipal Academy of Higher Education, Manipal 576104, India; deepthi.john1@learner.manipal.edu (D.M.J.); rajiv.md@manipal.edu (R.M.D.); km.nayak@manipal.edu (K.N.); sudheesh.pg@manipal.edu (S.P.G.); 2 Department of Mechatronics, Manipal Institute of Technology, Manipal Academy of Higher Education, Manipal 576104, India; shweta.vincent@manipal.edu; 3 Department of Information and Communication Technology, Manipal Institute of Technology, Manipal Academy of Higher Education, Manipal 576104, India; sameena.bp@manipal.edu; 4 Ignion, 08174 Barcelona, Spain; jaume.anguera@salle.url.edu; 5 Research Group on Smart Society, La Salle Engineering, Universitat Romon Llull, 08022 Barcelona, Spain; 6 Centre for Communications Technology, London Metropolitan University, London N7 8DB, UK; b.virdee@londonmet.ac.uk

**Keywords:** Sub-6 GHz, antenna array, MIMO, CMA, SAR, flexible

## Abstract

This paper presents a novel dual-band flexible antenna, uniquely designed and extended to array as well as MIMO configurations for the Sub-6 GHz band. The single-element monopole antenna features a modified rectangular radiator with two L-strips and a reduced ground plane, enabling a compact dual-band response. The proposed four-element, two-port MIMO configuration is extended from the 1 × 2 array antenna, achieving an overall dimension of 57 × 50 × 0.1 mm^3^, making it exceptionally compact and flexible compared to existing rigid and bulkier designs. Operating in the 3.6–3.8 GHz and 5.65–5.95 GHz bands, the antenna delivers a high gain of 5.2 dBi and 7.7 dBi, outperforming many designs in terms of gain while maintaining the superior isolation of >22 dB utilizing a defected ground structure (DGS). The design satisfies key MIMO diversity metrics (ECC < 0.05, DG > 9.99) and demonstrates low SAR values (0.0702/0.25 W/kg at 3.75 GHz and 0.175/0.507 W/kg at 5.9 GHz), making it highly suitable for wearable and on-body communication, unlike many rigid counterparts. Fabricated on a flexible polyimide substrate, the antenna addresses challenges such as size, bandwidth, isolation, and safety in MIMO antenna design. The performance, validated through fabrication and measurement, establishes the proposed antenna as a superior alternative to existing MIMO designs for compact, high-performance Sub-6 GHz 5G applications.

## 1. Introduction

The evolution of fifth-generation (5G) technology has driven the need for advanced antenna systems to meet diverse user requirements. Among the key enabling technologies for 5G communication, Sub-6 GHz communications offer superior coverage, straightforward implementation, and favorable propagation characteristics [1]. Multiband antennas within this spectrum have garnered a lot of attention due to their capacity to support numerous bands with a single antenna structure. However, designing compact multiband antennas for Sub-6 GHz poses a considerable challenge for antenna engineers. Critical parameters such as sufficient gain and a broader bandwidth must be maintained while achieving compactness, which is a challenging task given the increasing demand for miniaturized devices [2].

Despite this, achieving an extremely compact size for Sub-6 GHz antennas is constrained by the large operating wavelength (λ). Miniaturizing the aperture area can degrade the gain and efficiency, thereby impairing radiation performance. Although mm-wave bands offer potential, their implementation faces challenges such as limited coverage, severe fading, and high costs. Consequently, researchers have focused on developing compact multiband antennas for Sub-6 GHz with optimized performance characteristics [3,4].

Numerous methodologies have been explored to boost the performance of Sub-6 GHz antennas. For example, ref. [5] introduced a compact tri-band wearable antenna using an artificial magnetic conductor reflector, achieving reduced SAR and improved gain, albeit with a high volume. In [6], a multiband four-port antenna was designed with a unique lozenge-shaped DGS to improve isolation. While it achieved a low profile and enhanced isolation, its peak gain across all bands remained at just 4 dBi. Similarly, a slotted cavity-based antenna in [7] provided vertical polarization and omnidirectional characteristics, but its non-planar shape and limited bandwidth were drawbacks. In [8], a dual-band antenna utilized a meta-inspired network to enhance isolation and MIMO performance, but the design suffered from large size and low gain. A transparent antenna using metallic mesh, proposed in [9], offered decent bandwidth but had poor efficiency and fluctuating gain. In [10], a metasurface-based Sub-6 GHz antenna demonstrated a wider bandwidth and improved gain, but its isolation was limited to 15 dB and the design suffered from increased volume. Collectively, these studies highlight the difficulty of achieving wider bandwidth, higher gain, enhanced isolation, and compactness simultaneously in Sub-6 GHz antennas.

To overcome these challenges, researchers have explored array antennas, which are well-established for enhancing gain [11,12]. For instance, ref. [13] proposed a 1 × 4 MIMO antenna array for 5G terminals using a corporate feed with elements spaced less than 0.5λ apart, resulting in a directive radiation pattern and low sidelobe levels. However, its large size and semi-flexible substrate made it unsuitable for wearable applications. In [14], an array antenna with frequency-selective structures achieved a stable 12 dBi gain, but its rigid substrate and large volume posed limitations. A leaf-shaped 1 × 4 corporate feed array in [2] achieved a gain improvement of up to 3.95 dB but lacked flexibility due to its rigid FR4 substrate. These studies reveal a gap in the development of high-gain, Sub-6 GHz flexible array antenna structures.

To address the demand for high data rates and low latency, MIMO antennas have emerged as a promising solution. MIMO structures enhance the channel capacity by integrating multiple antenna elements without increasing the transmission power [15]. While several flexible MIMO antennas have been proposed for the 5G mid-band [16,17,18], there is a lack of flexible MIMO array antenna designs, particularly for Sub-6 GHz bands. Although [13] extended its 1 × 4 array to a MIMO configuration, the design lacked multiband functionality, remained semi-flexible, and had a large volume.

To bridge these gaps, a dual-band two-port Sub-6 GHz MIMO array antenna is proposed in this brief article. This design features a unique DGS that provides a minimum isolation of better than 22 dB across both operating bands (3.6–3.8 GHz and 5.65–5.95 GHz). The antenna exhibits high flexibility and compact dimensions of 57 × 50 × 0.1 mm^3^, with a modified rectangular radiator incorporating L-strips and a reduced ground plane. In the first and second bands, it attains a radiation efficiency and peak gain of 5.2 dBi/92% and 7.7 dBi/91%, respectively. Additionally, the antenna satisfies the MIMO diversity metrics necessary for reliable communication and offers low SAR values. The resonance behavior of the antenna is analyzed using characteristic mode theory, and the design addresses the key limitations in current Sub-6 GHz antenna research.

## 2. Single-Element Antenna

This section elaborates on the design of the single-element dual-band Sub-6 GHz antenna. The design procedure of the antenna is discussed in detail, highlighting the evolution stages followed by the parametric analysis of different design parameters.

### 2.1. Design Methodology

**Figure 1:**
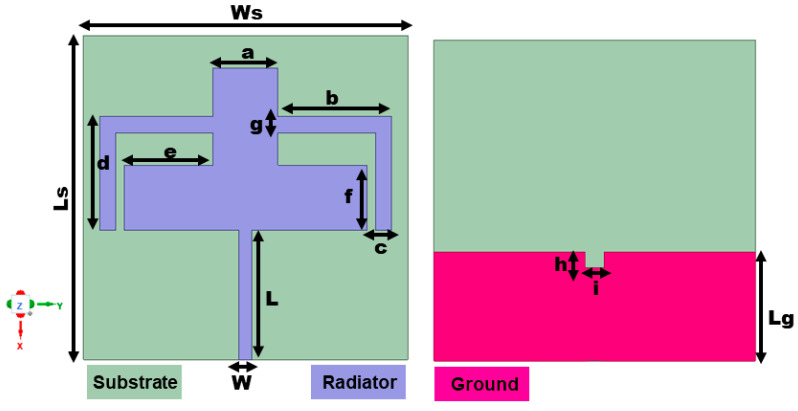
Single-element design configuration.

The schematic of the suggested dual-band antenna is given in Figure 1. The antenna design makes use of a flexible polyimide substrate with a dissipation factor of 0.008 and a dielectric value of 3.5. The overall physical values of the suggested antenna are 20 × 20 × 0.1 mm^3^. The values of the design parameters are tabulated in Table 1. The table provides information about the single-element antenna dimensions including feedline width and length, stub length and width, ground length, and dimensions of slit incorporated in the ground plane. In terms of impedance bandwidth, the suggested antenna has a dual-band response that spans 3.68–3.85 GHz (resonance at 3.75 GHz) and 5.84–6.06 GHz (resonance at 5.9 GHz), respectively.

**Figure 2:**
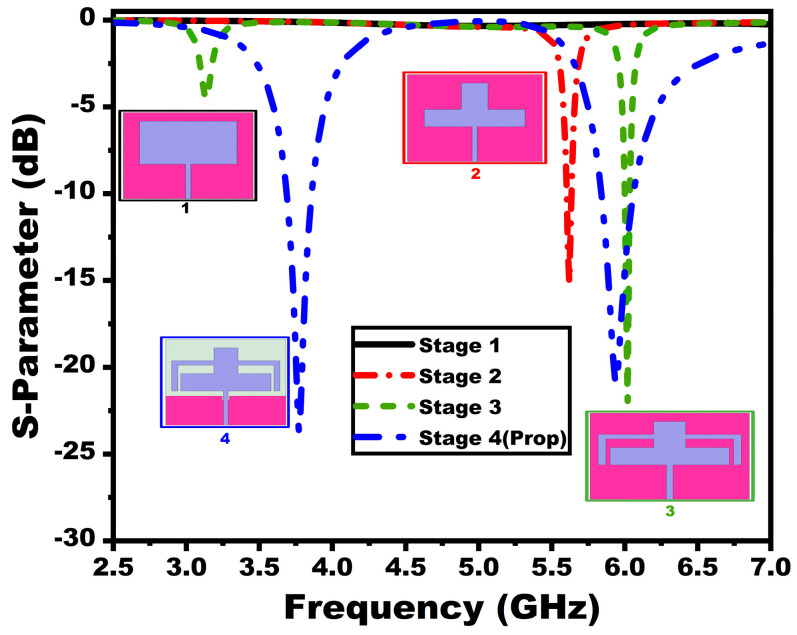
Reflection coefficient corresponding to different design stages.

**Figure 3:**
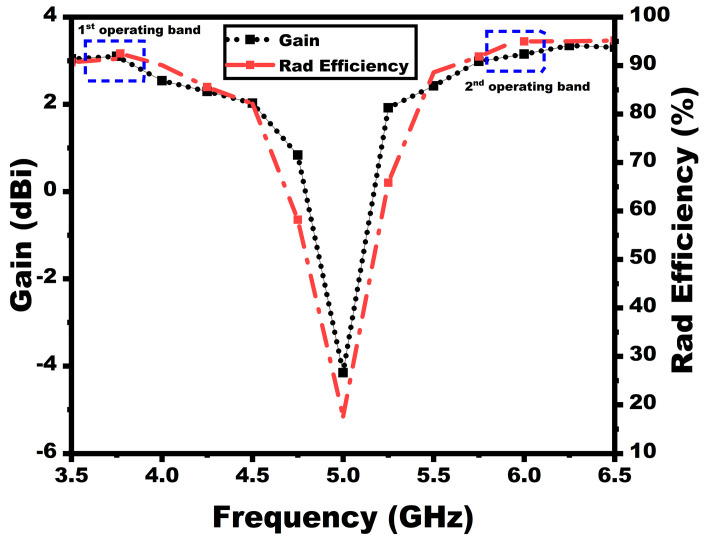
Simulated gain and efficiency plots.

The suggested antenna has progressively evolved in four stages to achieve the prescribed dual bands. The antenna modeling starts with a rectangular patch antenna, which provides scattering parameters below −10 dB. The second stage incorporates two rectangular slits in the radiator. The antenna operates at 5.62 GHz in this stage with a very low bandwidth of 5.6–5.64 GHz. To provide a dual-band response, the third stage is introduced with two L-shaped strips in the radiator. This stage showed two bands below −6 dB from 3.09–3.19 GHz and 5.9–6.04 GHz. Although the antenna gives a two-band response, the bandwidth is very low. To incrementally increase the antenna bandwidth with a dual-band response below −10 dB, the antenna ground is made partial with a rectangular cut towards its center. Reducing the antenna ground is a well-known technique for improving the bandwidth by reducing the antenna quality factor. The rectangular cut, on the other hand, provides better impedance matching. The suggested last stage exhibits a dual-band operation with resonating at 3.75 GHz and 5.9 GHz, respectively, and spans from 3.68–3.85 GHz to 5.84–6.06 GHz. Figure 2 depicts the suggested single-element antenna’s reflection properties at various stages. The antenna offers gain and efficiency of 3 dBi/90% and 3.6 dBi/93% for the first and second bands, as illustrated in Figure 3.

### 2.2. Parametric Study

**Figure 4:**
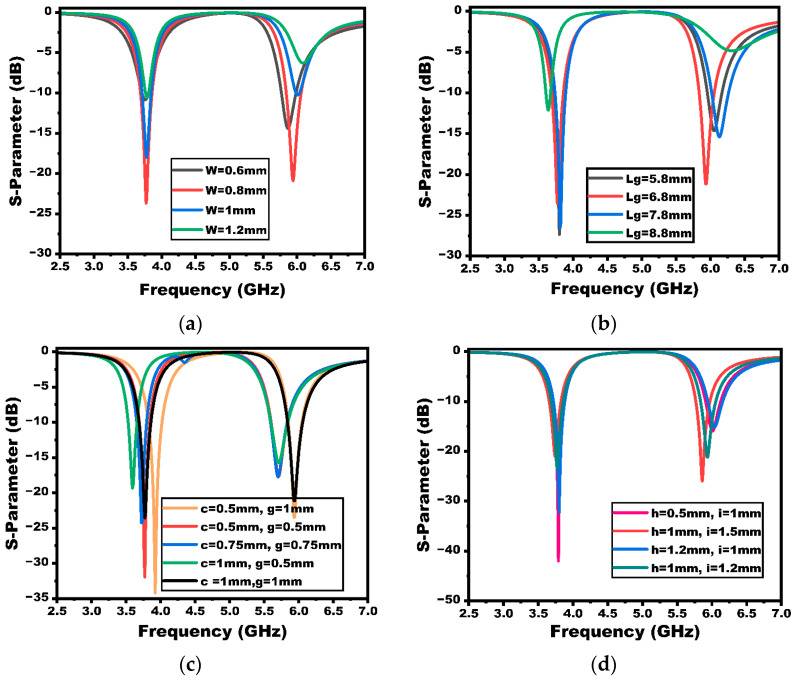
Parameter optimization of different antenna design variables: (**a**) width of the feedline, (**b**) ground plane length, (**c**) length and width of the strip, and (**d**) length and width of the ground plane rectangular slot.

The antenna dimensions are optimized using parametric analysis, where the major antenna parameters are analyzed to achieve the desired dual-band response. The feed width (W), ground plane length (Lg), thickness of L-shaped strips, and dimensions of the rectangular cut in the ground are optimized. The corresponding reflection coefficients are compared as shown in Figure 4. The optimal values of the design parameters are finally chosen, providing the desired bandwidth. As exhibited in the figure, the feed width is chosen to be 0.8 mm. Improving the antenna bandwidth is greatly impacted by reducing the ground length. The size of the ground is optimized, and a value of 6.8 mm is chosen which provides a wider bandwidth and requisite frequency of resonance. The widths of L-strips have been a crucial parameter for achieving the desired frequency bands. The width of each branch of the L-strip is chosen as 1 mm, as provided in the figure. The width and height of the rectangular cuts in the ground plane have contributed to good impedance matching. These parameters have also been crucial in achieving a wider bandwidth and frequency of operation for the second band. The height and width of the rectangular cuts are optimized to a value of 1 mm and 1.2 mm, respectively.

## 3. 1 × 2 Array Antenna

This section elaborates on the development of a 1 × 2 array antenna that provides dual-band response and high gain for mid-band applications.

### 3.1. Design Methodology

**Figure 5:**
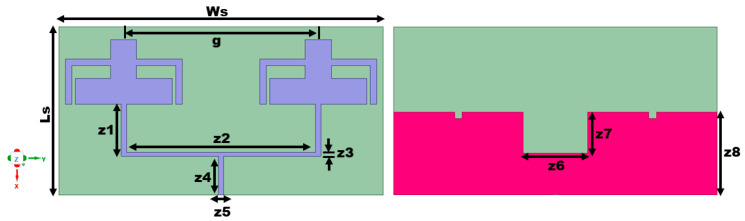
The configuration of the 1 × 2 array antenna.

As depicted in Figure 5, the single-element antenna is modified to a 1 × 2 array antenna where the single feed feeds the power. The antenna elements are kept apart at a center-to-center distance of 0.375λ (less than λ/2) to avoid inter-element coupling (λ is computed corresponding to the lowest operating frequency). The spacing between the neighboring elements of the array is optimized progressively to have minimal degradation of its performance. Finally, a value of 0.375λ is chosen as an optimal spacing that provides satisfactory gain, scattering parameters, and radiation efficiency. The proposed array antenna has an overall dimension of 26 × 50 × 0.1 mm^3^. The main aim of designing the array is to boost the gain of the single-element antenna. As discussed in the earlier sections, the antenna gain is not high in Sub-6 GHz bands, when compared to the higher frequencies like the millimeter wave bands [2]. This is due to the comparatively higher wavelength of this band than the higher-frequency bands. Figure 5 shows the structure of the suggested 1 × 2 array antenna. In the proposed array structure, the power is fed using a corporate feed technique where the power divider involved is optimized to ensure that the same amount of power is delivered to each element to achieve efficient radiation characteristics. The dimensions of the 1 × 2 array are tabulated in Table 2. The dimensions of the transmission line width that correspond to 50 ohms (z5) and 100 ohms (z3) are included in the table, calculated using Equation (1).

The width of the transmission line of the array antenna can be estimated using the equation to guarantee an equal power distribution [13]:

(1) $$w_{z_{o}}=\left(\frac{377}{Z_{c}\sqrt{\varepsilon_{r}}}-2\right)h$$

where $Z_{c}$ defines the transmission line’s characteristic impedance, and $\sqrt{\varepsilon_{r}}$ defines the relative permittivity.

**Figure 6:**
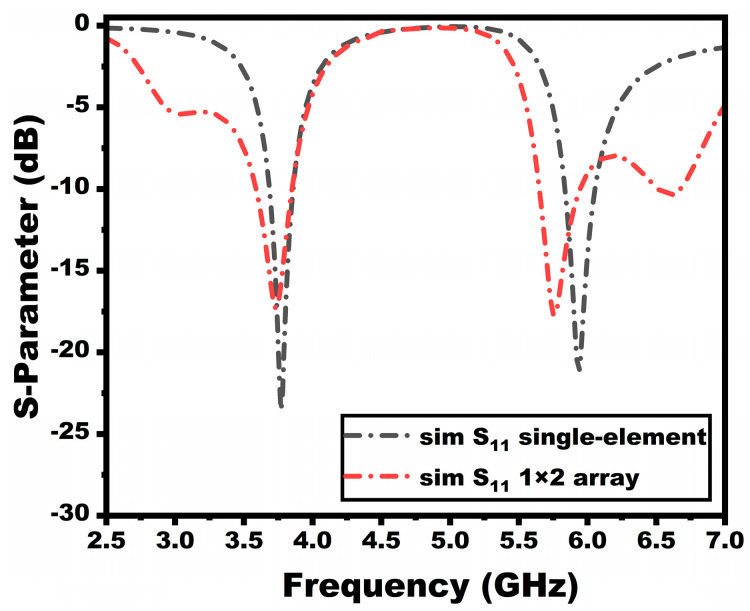
Reflection coefficient comparison between the array and single-element antenna.

The reflection coefficients of the proposed single-element antenna and array antenna are compared in Figure 6. As discussed earlier, the single element has a bandwidth of 3.68–3.85 GHz and 5.84–6.06 GHz. The array antenna, on the other hand, provides bandwidth from 3.59–3.84 GHz to 5.65–5.95 GHz, resonating at 3.75 GHz and 5.76 GHz, respectively. In the array antenna design, there is a slight resonance frequency variation towards the second band due to the alteration of the ground. However, the antenna still operates in the desired frequency range of operation.

### 3.2. Measured Results and Discussion

**Figure 7:**
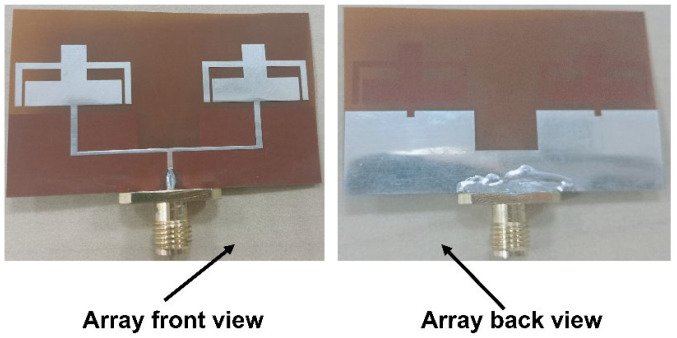
Array antenna fabricated prototype.

**Figure 8:**
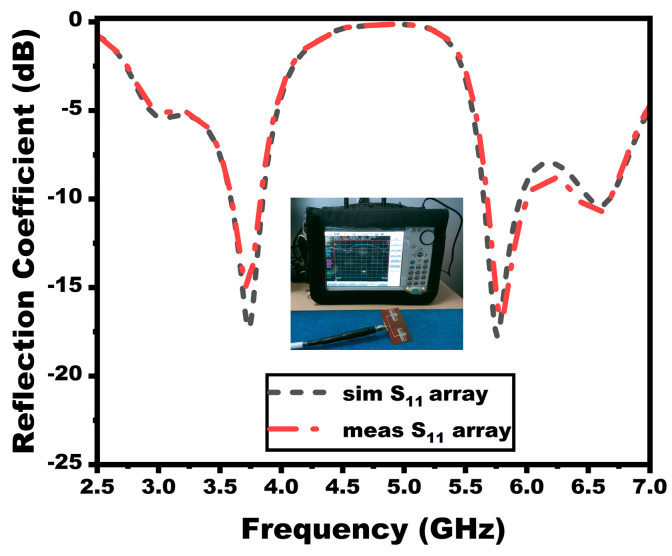
Reflection coefficients of the array antenna.

As is apparent from Figure 7, the array antenna is constructed on a flexible polyimide substrate. The Anritsu S820E VNA (Atsugi, Japan), which operates in the frequency range of 1 MHz to 40 GHz, is used to measure the scattering parameters of the suggested antenna. Figure 8 shows the reflection coefficient between the simulated and evaluated findings of the suggested array antenna. The array antenna provides a dual-band response with a 3.59–3.83 GHz to 5.68–6.01 GHz operating range.

**Figure 9:**
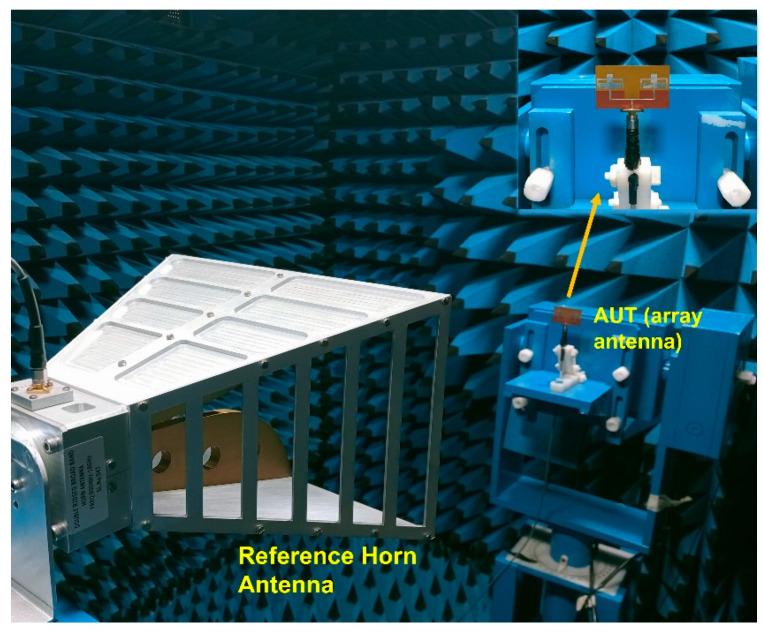
Array antenna radiation pattern measurement.

**Figure 10:**
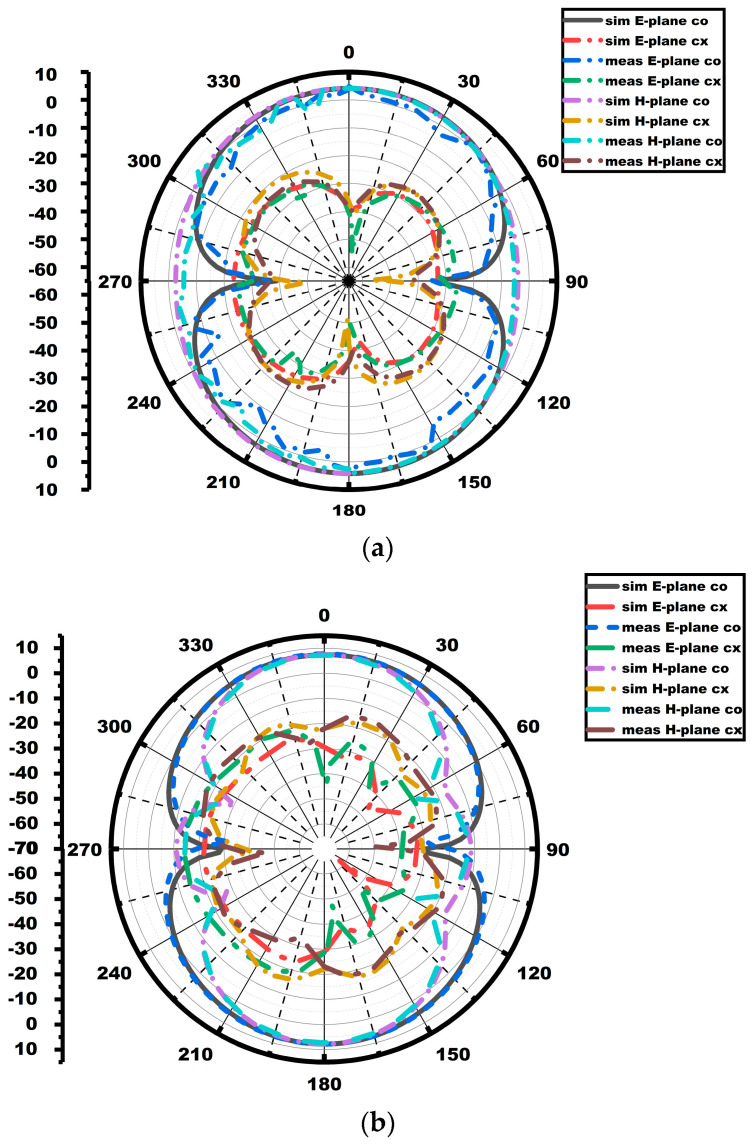
Array radiation patterns at (**a**) 3.75 GHz and (**b**) 5.76 GHz.

The radiation characteristic evaluation of the array antenna using the anechoic chamber is given in Figure 9. A double-ridged horn antenna (18 GHz) is utilized as a reference antenna while the fabricated array antenna is used as the AUT (antenna under test). The 2D radiation patterns (co- and cross-polarization) at the major E and H planes at the resonant frequencies 3.75 and 5.76 are given in Figure 10.

**Figure 11:**
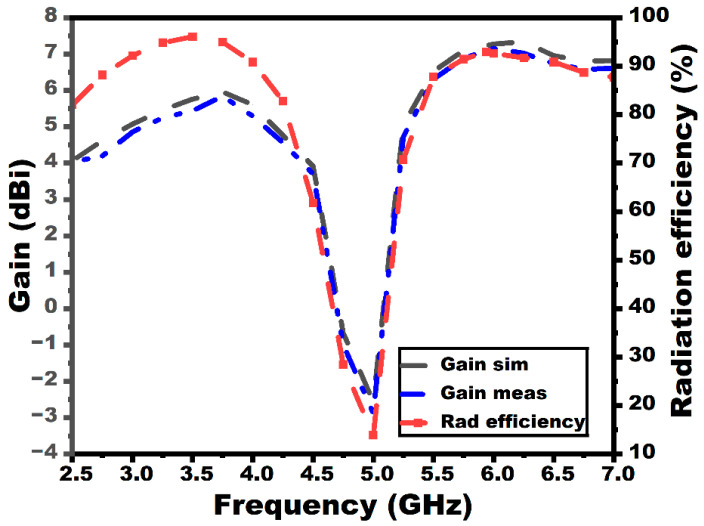
Gain and radiation efficiency plots of the array antenna.

The proposed array antenna exhibits bidirectional patterns in the E-plane and quasi-omnidirectional patterns in the H-plane, as portrayed in the figure. Figure 11 displays the gain as well as radiation efficiency of the array antenna. The array antenna exhibits a gain and efficiency of 4.8 dBi/91% and 7 dBi/90%, respectively, for the first and the second operating bandwidth. The gain graphs in Figure 11 demonstrate that the array antenna’s gain outperforms the single-element antenna.

## 4. MIMO Array Antenna

This section explains the design and development of the four-element two-port MIMO array antenna.

### 4.1. Methodology

**Figure 12:**
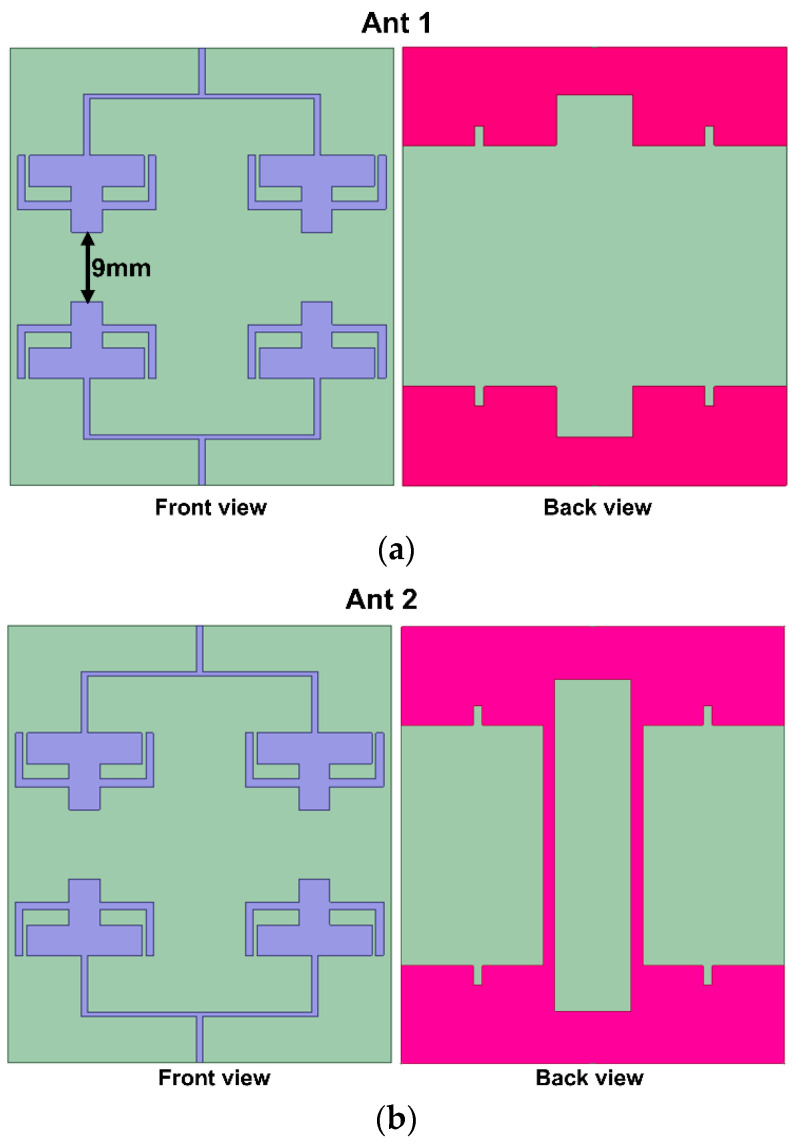
Evolution stages of the MIMO configuration (**a**) without DGS and (**b**) with DGS (ground connected with stubs, as proposed).

**Figure 13:**
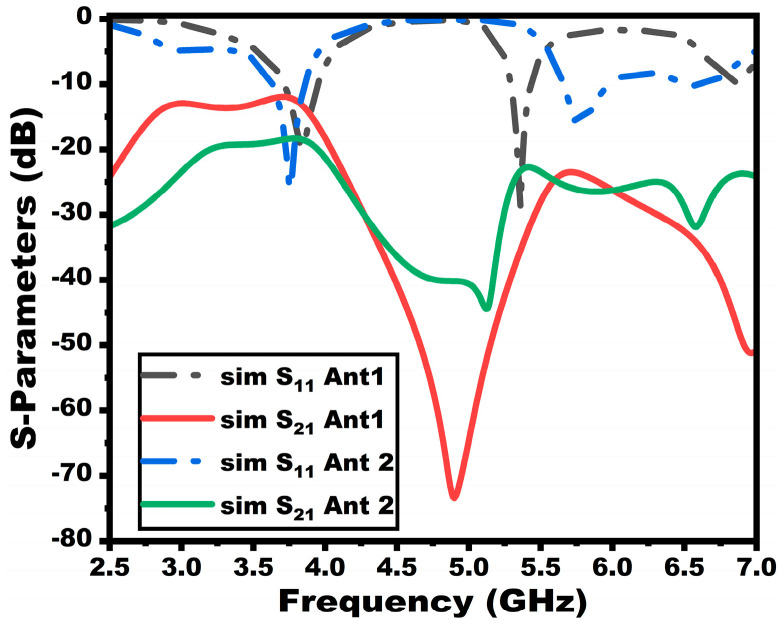
Simulated scattering properties of different MIMO design stages.

The proposed 1 × 2 array antenna, discussed in Section 3, is extended to a two-port MIMO array antenna, as shown in Figure 12. Initially, at stage 1 (Ant 1), the MIMO configuration is formed by mirroring the 1 × 2 array antenna towards the *x*-axis at an element spacing of 9 mm (less than one-fourth of the wavelength) as shown in Figure 12a. The second stage (Ant 2) shows the final proposed MIMO antenna incorporating two vertical stubs that form the entire connected ground and DGS decoupling structure. Figure 13 displays the simulated scattering properties of the MIMO configuration’s evolution stages. From the figure, the reflection coefficient of the first evolution stage has a reduced bandwidth and frequency of operation.

**Figure 14:**
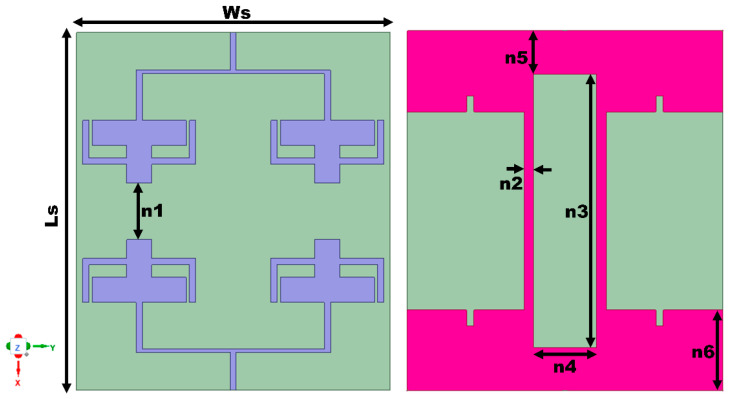
Schematic of the MIMO array antenna. All values in mm: Ls = 57, Ws = 50, n1 = 9, n2 = 1.5, n3 = 43.4, n4 = 10, n5 = 6.8, and n6 = 12.8.

Moreover, the minimum isolation throughout the entire bandwidth is less than 15 dB for the first band. However, the final proposed stage (Ant 2) provides an enhanced 22 dB isolation for both of the operating frequency ranges. The rectangular stubs that are connected to the ground create an alteration in the current path, thereby canceling the coupling current and enhancing isolation. The volume of the proposed MIMO antenna is 57 × 50 × 0.1 mm^3^. Figure 14 presents the dimensions and final configuration of the suggested MIMO antenna.

### 4.2. Bending Analysis of the Proposed Antenna

**Figure 15:**
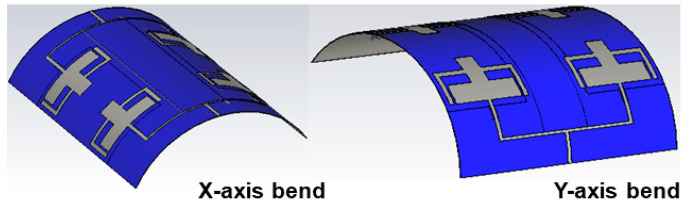
Bending analysis of the antenna in the *x*-axis and *y*-axis bends.

**Figure 16:**
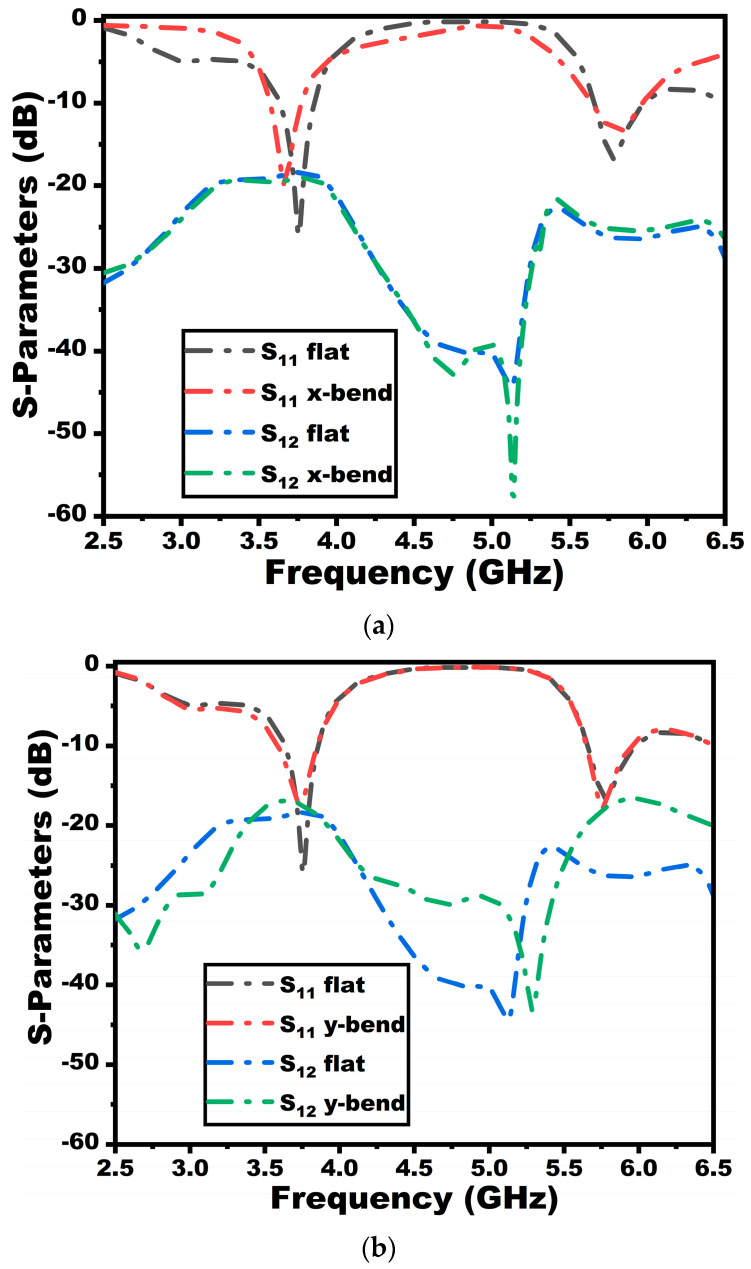
S-parameters of the suggested antenna under bending environment (**a**) *x*-axis bend, and (**b**) *y*-axis bend.

One of the prominent analyses involving the design of the antenna for wearable scenarios is the bending analysis. The performance of the antenna changes when the antenna is kept in a bent position according to the human body’s curvatures [6]. To study the performance of the antenna in a bending environment, the proposed antenna is analyzed with two different bending conformations (*x*-axis and *y*-axis bends) with a 50 mm radius of bend, as portrayed in Figure 15. The analysis is performed using cylindrical bends available in the CST Studio EM tool. The S-parameters of the antenna are observed under both the bending axis and compared with the performance under flat conditions, as evaluated in Figure 16. It is evident that under both scenarios, there is a slight shift in the resonance in the first band, whereas the second band does not show any variations. The bandwidth in both bands is also retained, showing the antenna’s applicability in wearable applications.

### 4.3. Characteristic Mode Analysis

The characteristic mode theory has been a popular antenna analysis tool for understanding any antenna structure’s operating behavior. CMA uses information on different resonant modes, facilitating the understanding of the significant modes that influence the antenna’s resonance. The CMA calculates that the whole antenna current is the vector addition of the nth eigen current $J_{n}$ and modal weighting coefficient $\beta_{n}$ that is derived from the eigenvalue $\lambda_{n}$, which is denoted by Equation (2) [19].

(2) $$J=\sum_{n}\beta_{n}J_{n}\;$$

The CM can be estimated from $\lambda_{n}$ using Equation (3).

(3) $$X\;\left(J_{n}\right)=\lambda_{n\;}R\left(J_{n}\right)$$

where *R* denotes the real part of the impedance matrix Z.

**Figure 17:**
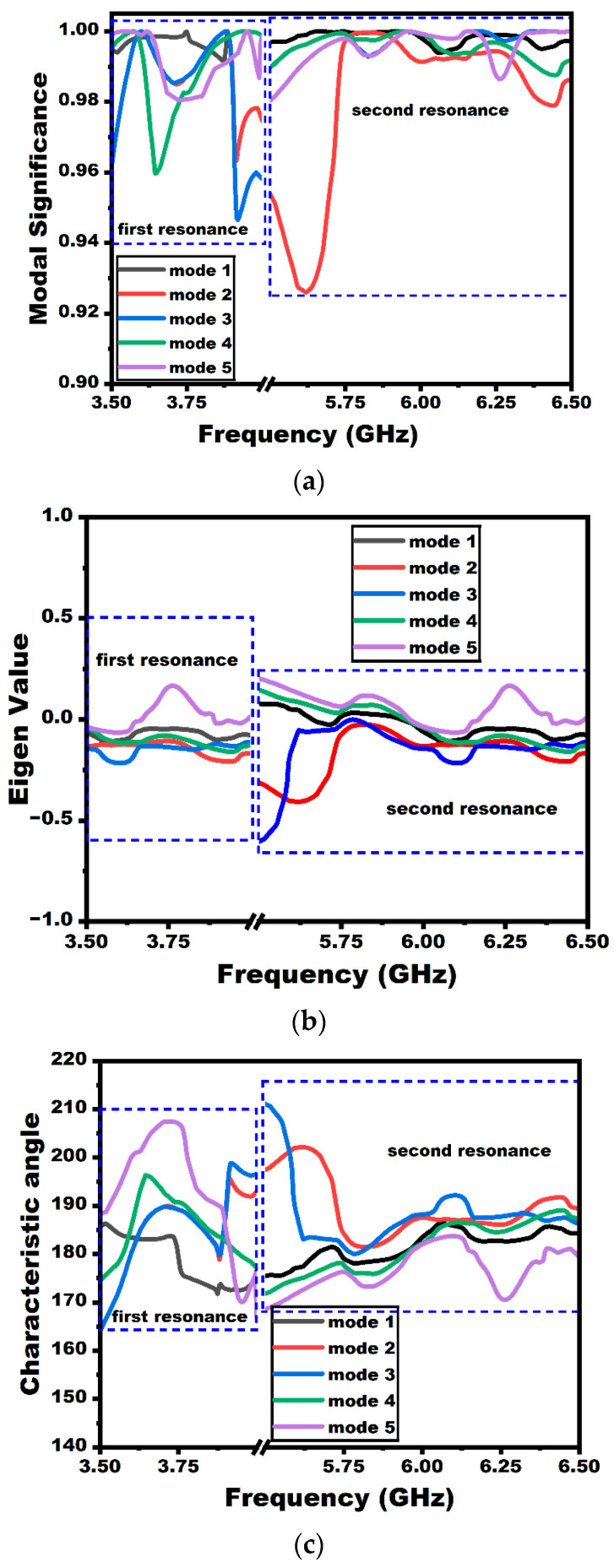
CMA analysis: (**a**) modal significance, (**b**) eigenvalue, and (**c**) characteristic angle.

The eigen-based parameter’s modal significance (MS) and characteristic angle (CA) are investigated in the study to understand the resonance behavior of the proposed antenna. The CA determines the corresponding field current phase delays. In contrast, the modal significance indicates the normalized current mode value, where a mode resonates when the MS value is near unity. In other words, the particular mode resonates when the eigenvalue is zero or the CA is close to 180°. Figure 17 shows the CMA parameters of the suggested MIMO antenna at both resonances. The study uses the first five fundamental modes (modes 1–5) to examine the antenna’s resonance behavior. Initially, let us consider the first resonance. From the figure, the first resonance of the antenna is highly influenced by mode 1, since the modal significance approaches unity and the eigenvalue of that particular mode is zero. This can be further confirmed by the CA value, where the value approaches 180° for the first mode. The individual current modes contribute to the resonance when the MS value is more than 0.707 [20]. Even though all the other modes contribute to a value higher than 0.707, mode 1 is the most significant mode that helps in radiating energy and contributes much to the first resonance. In addition, it can be deduced from the figure that every mode contributes to the second resonance of the suggested antenna, which has an MS greater than 0.707. However, it is visible that modes 1 and 4 are more significant and contribute to the second resonance with an MS value equal to unity near the second antenna resonance and also over the entire second band of operation. The CA value also approaches 180° for these two modes, indicating the antenna’s radiation.

The CMA has been investigated in this study to examine the resonant behavior and how the individual modes of the antenna have contributed to both of the antenna’s resonances.

**Figure 18:**
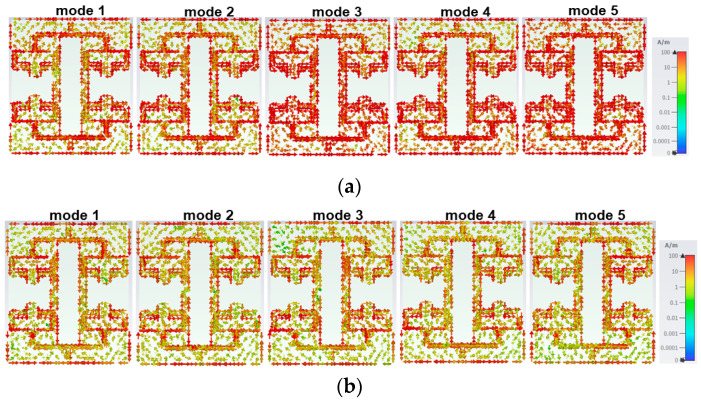
Surface current distribution of CM modes: (**a**) first resonance and (**b**) second resonance.

The surface current distribution of all the characteristic modes (represented by mode 1 to mode 5) for both resonance frequencies is displayed in Figure 18 to enhance the clarity. The significant individual mode current contributes to achieving the desired radiation properties with bidirectional and omnidirectional patterns in the principal E and H planes, respectively.

### 4.4. Results and Discussion

**Figure 19:**
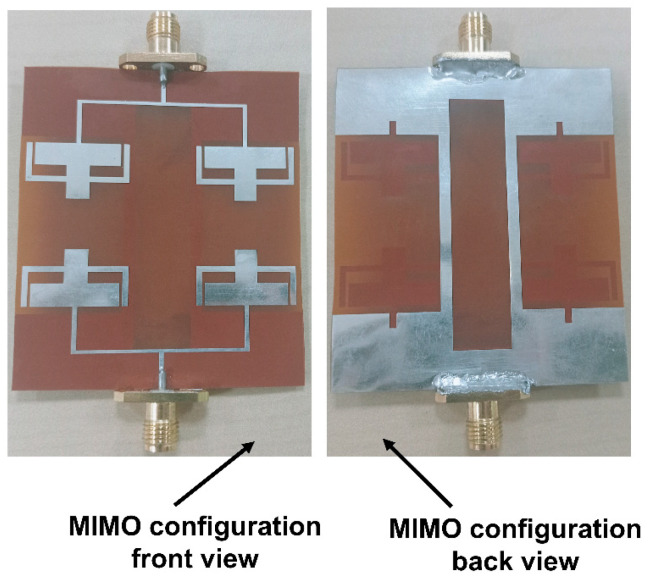
Fabricated prototype of the MIMO configuration.

**Figure 20:**
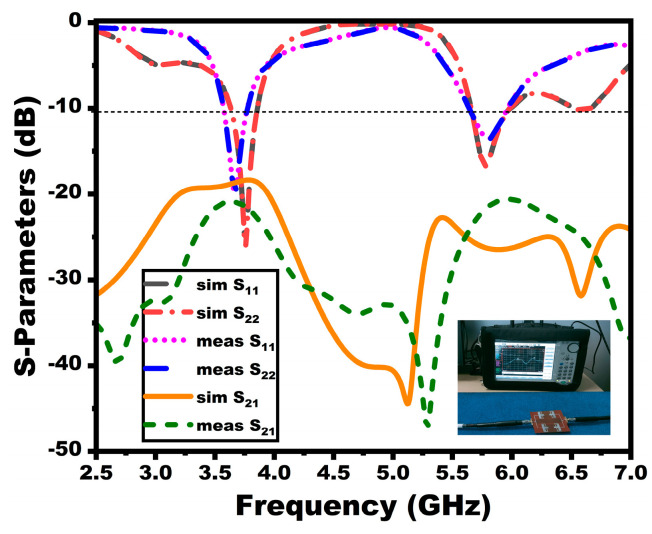
Simulated and measured scattering parameters of the MIMO configuration.

The suggested MIMO antenna prototype is provided in Figure 19. The antenna holds a measured bandwidth of 3.6–3.8 GHz and 5.65–5.95 GHz with a resonance of 3.75 GHz and 5.9 GHz, respectively. There is a negligible shift in the first resonance of the proposed antenna due to cable losses. Figure 20 displays the proposed MIMO array antenna’s measured scattering properties.

**Figure 21:**
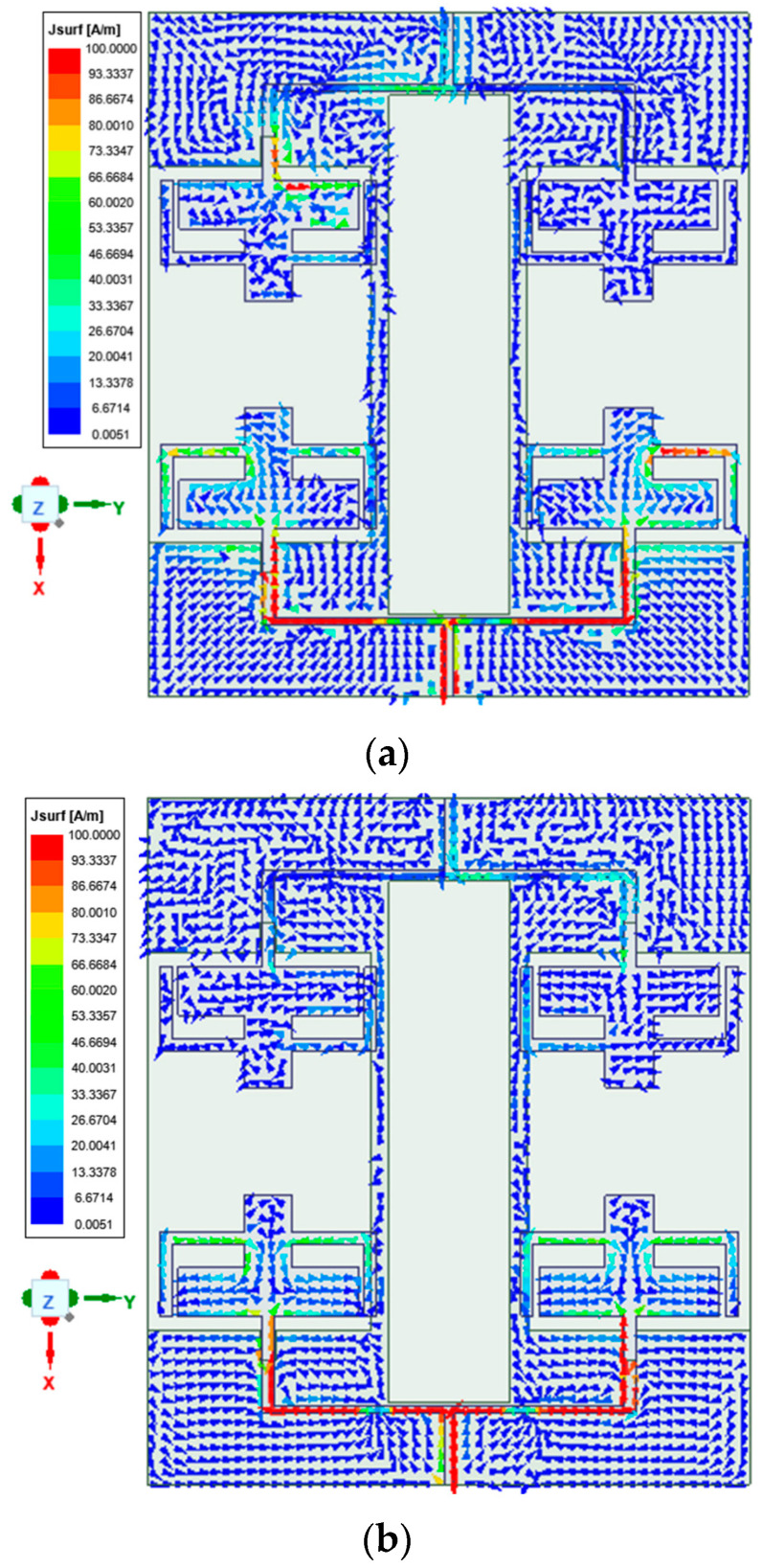
Vector current distribution: (**a**) 3.75 GHz; (**b**) 5.9 GHz.

From Figure 20, the presented antenna offers enhanced isolation in the operating bands. The mechanism of the decoupling structure can be inferred from the current vector distribution plots portrayed in Figure 21 at both resonant frequencies. It is investigated by exciting port 1 and terminating port 2. From the current distribution, it is seen that the maximum current is concentrated on elements in the excited port and the decoupling structure. On the other hand, there is a trivial current concentration in the nearest elements. The current concentration in the decoupling structure shows that the current direction is opposite to each other, indicating that the coupling current cancels out each other, thereby offering enhanced isolation.

**Figure 22:**
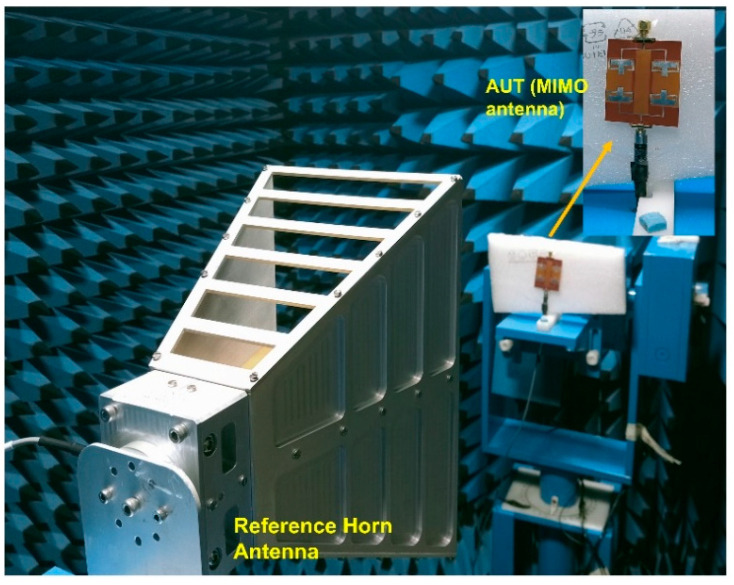
Radiation characteristic measurement.

**Figure 23:**
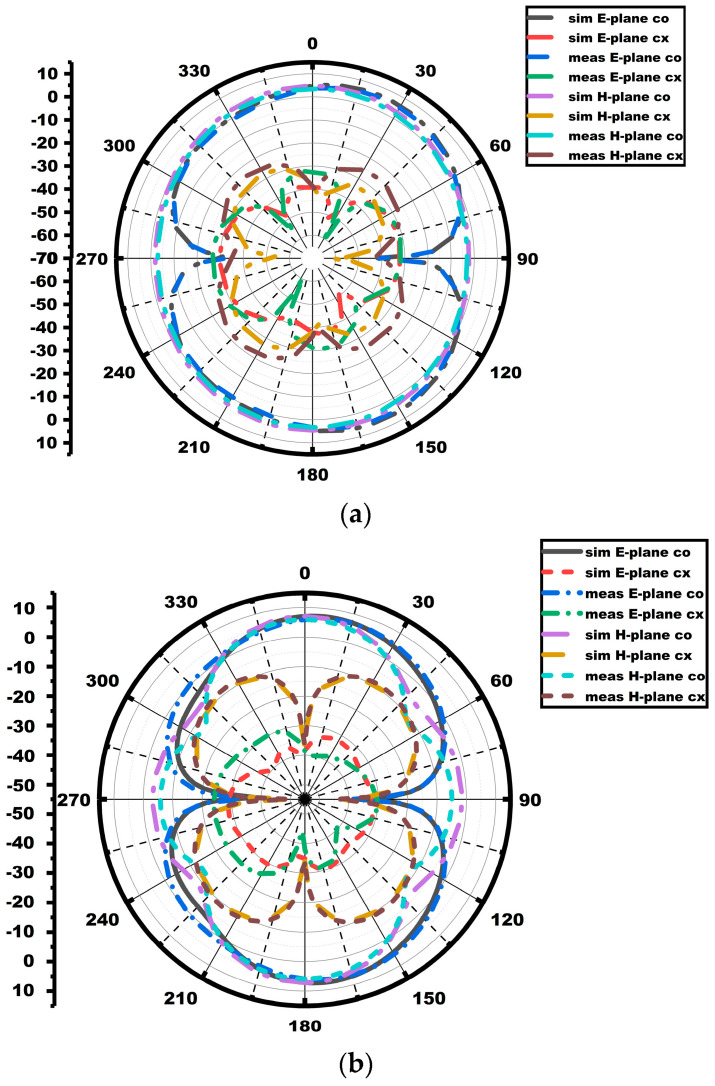
Radiation patterns of MIMO antenna: (**a**) 3.75 GHz; (**b**) 5.9 GHz.

**Figure 24:**
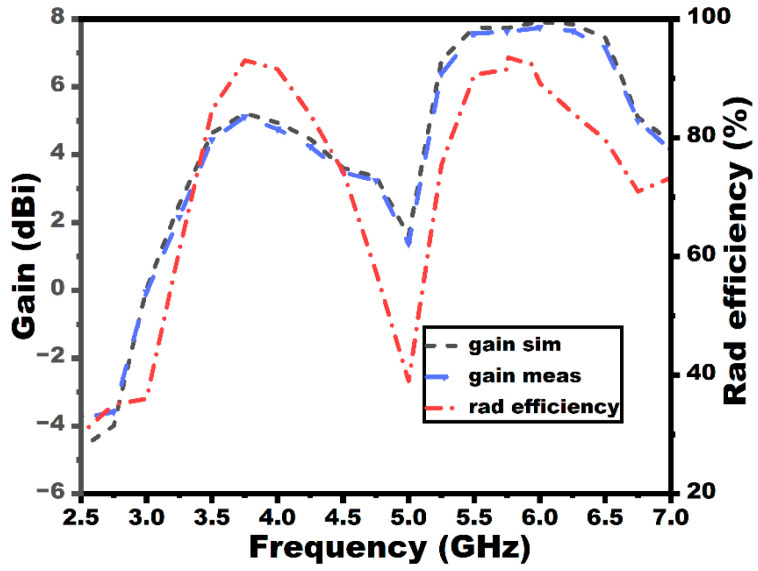
Gain and efficiency plots of the MIMO configuration.

The radiation characteristic measurement of the MIMO array antenna in the chamber is shown in Figure 22. A double-ridged horn is utilized as the reference antenna, and the proposed MIMO array antenna is kept as the antenna under test (AUT), as seen from the figure. The radiation characteristics in both E and H planes (co- and cross-polarization) of the MIMO array antenna are depicted in Figure 23. The radiation pattern gives significant variation between the co- and cross-polarization. The proposed antenna shows the bidirectional and omnidirectional types of characteristics in the E and H planes, respectively. The max gain and radiation efficiency plots of the suggested antenna are provided in Figure 24. The antenna exhibits a gain value of 5.2 dBi/92% and 7.7 dBi/91% at the first and second resonances, as given in the figure.

**Figure 25:**
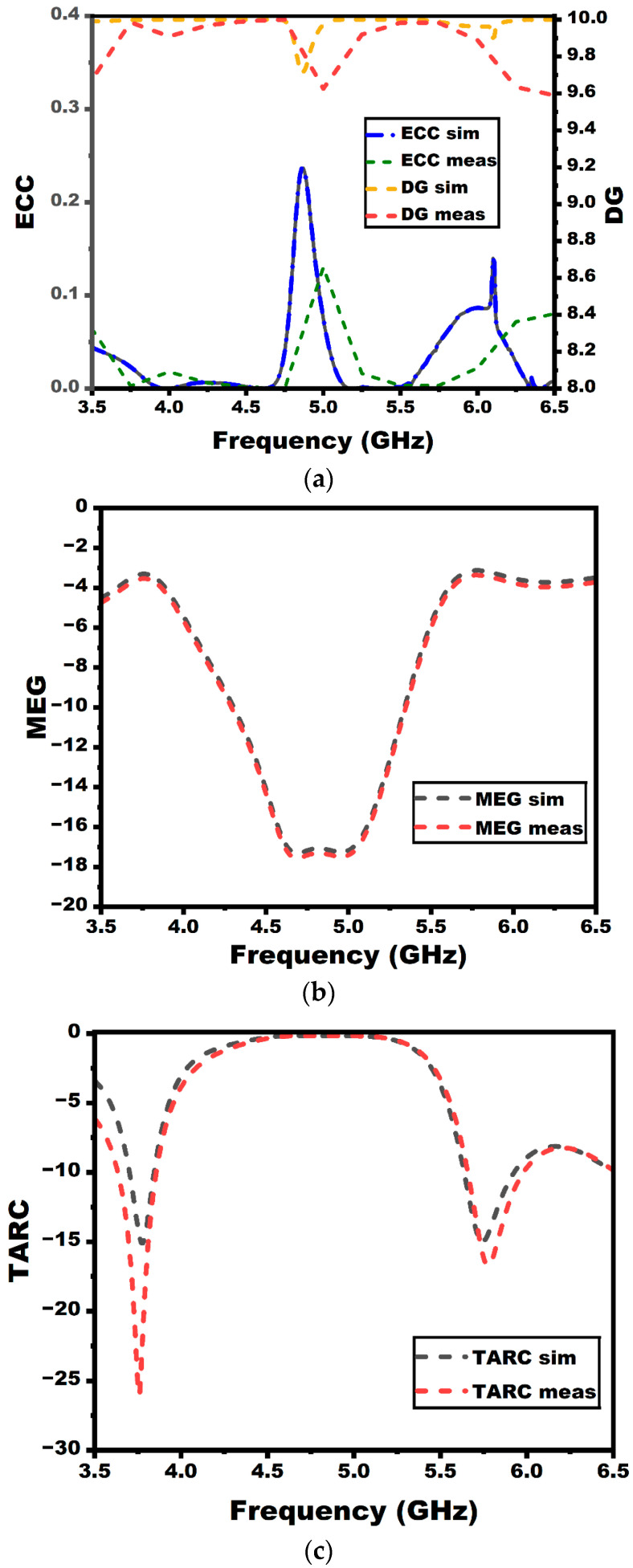
MIMO diversity parameters: (**a**) ECC and DG, (**b**) MEG, and (**c**) TARC.

The diversity parameter estimation of the suggested antenna is given in Figure 25a–c. The envelope correlation coefficient (ECC) evaluates the correlation of the individual elements of the MIMO antenna. Diversity gain (DG) is the parameter that indicates the transmission capacity of the antenna when the diversity system is utilized. Equation (4) can be used to determine the ECC from the far-field patterns [21]. DG can be derived from ECC using Equation (5). The proposed antenna holds ECC and DG values of <0.05 and >9.99 dB, respectively, for both bands. The mean effective gain (MEG), which can be calculated using Equation (6), compares the energy obtained by an isotropic antenna to a MIMO antenna in a fading environment. The suggested antenna gives a MEG value of −3 dB over the bands. The proposed antenna’s total return loss characteristics can be estimated using the total active reflection coefficient (TARC). Equation (7) computes the TARC of the proposed MIMO array antenna.

(4) $${ECC}_{\rho }=\frac{{\left|\iint_{4\pi }\left[E_{1}\left(\theta ,\phi \right).E_{2}\left(\theta ,\phi \right)d\Omega \right]\right|}^{2}}{\iint_{4\pi }{\left|{E_{1}\left(\theta ,\phi \right)|}^{2}d\Omega \iint_{4\pi }\right|E_{2}\left(\theta ,\phi \right)|}^{2}d\Omega }$$

(5) $$DG=10\sqrt{1-{ECC}^{2}}$$

(6) $${MEG}_{i}=0.5\left(1-\Sigma_{j=1}^{M}\left|S_{ij}\right|\right)$$

(7) $$TARC=\sqrt{\frac{\left(|S_{11}+S_{12}{|}^{2})+(|S_{21}+S_{22}{|}^{2}\right)}{2}}$$

## 5. SAR Analysis of the MIMO Array Antenna

**Figure 26:**
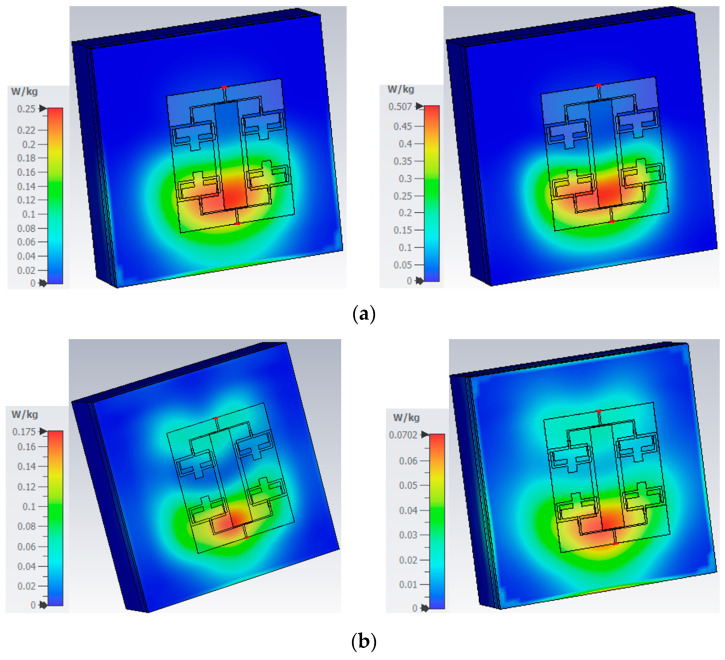
SAR values at (**a**) 3.75 GHz and (**b**) 5.9 GHz.

One of the major applications of the flexible antenna is as a wearable application, where the antennas are positioned near the human body. The essential parameter to be evaluated is the specific absorption rate (SAR) value that accounts for the maximum level of radiation absorbed by the human body. According to international norms, this is restricted to 1.6 W/kg and 2 W/kg for every 1/10 g of human tissue, respectively. To analyze the SAR value of the proposed antenna, a three-layer tissue model is formed using the CST studio suite. The model comprises muscle, fat, and skin layers that have different dielectric properties at different frequencies. The SAR value at both the resonances for 1/10 g is estimated, as given in Figure 26. The tissue dielectric properties at these frequencies are used from the ITIS database [22]. The antenna provides an SAR of 0.0702/0.25 W/kg and 0.175/0.507 W/kg for 1/10 g at 3.75 GHz and 5.9 GHz, respectively. The suggested antenna is ideally suited for on-body applications, as seen by the low SAR values.

**Figure 27:**
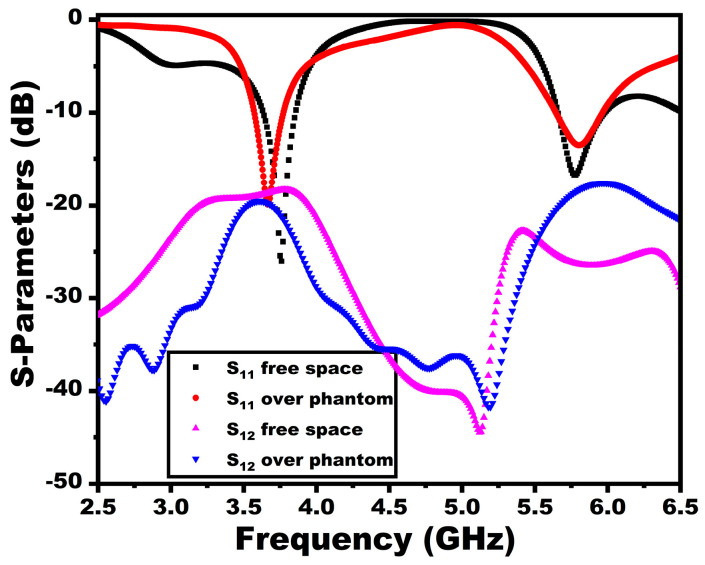
Reflection coefficient comparison in free space and over the phantom.

**Figure 28:**
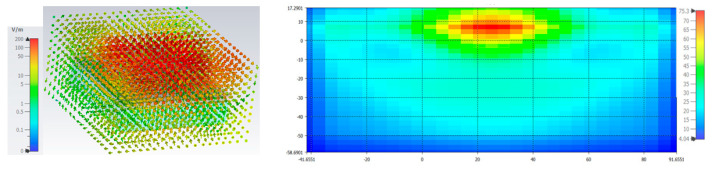
Electric field and SAR with field exposure estimation.

The scattering properties of the proposed antenna over the phantom are displayed in Figure 27. As depicted in the figure, there is a slight shift in the resonant frequency of the antenna towards the first band due to the dielectric properties of the tissues. However, the antenna still maintains the operational bandwidth, as indicated in the figure. The E-field at 3.77 GHz and the SAR value accompanied by the internal field along the depth region are given in Figure 28.

## 6. Comparative Study

Table 3 demonstrates a comparison of the proposed flexible MIMO array antenna with similar works currently in existence in the literature for the Sub-6 GHz band. The literature shows minimal recent works in the Sub-6 GHz band, where an antenna array is extended to a MIMO configuration. In terms of its small size, bandwidth, and flexibility, the suggested antenna outperforms those presented in the current literature, according to the comparative table given below. The proposed antenna also offers a dual-band response with better isolation, good MIMO characteristics, and a reduced SAR value that falls below the international values.

## 7. Conclusions

The proposed dual-band flexible four-element two-port MIMO antenna array designed for the Sub-6 GHz band offers significant advantages in terms of size, flexibility, and performance. The single-element monopole antenna, featuring a modified rectangular radiator with L-strips and a reduced ground plane, is fabricated on a flexible polyimide substrate, achieving a compact size of 57 × 50 × 0.1 mm^3^. It is further demonstrated that extending the single element to a 1 × 2 array and subsequently to a four-element MIMO configuration improves the gain (5.2 dBi and 7.7 dBi for the two bands), bandwidth (3.6–3.8 GHz and 5.65–5.95 GHz), and isolation (>22 dB), outperforming many existing designs that often lack flexibility or provide lower isolation.

The suggested antenna achieves a good MIMO diversity performance, with ECC < 0.05, DG > 9.99, MEG ~ −3 dB, and TARC < −10 dB, ensuring reliable operation in diverse environments. Furthermore, the low SAR values (0.0702/0.25 W/kg at 3.75 GHz and 0.175/0.507 W/kg at 5.9 GHz) confirm its suitability for wearable and on-body applications, addressing limitations seen in rigid and bulkier counterparts.

The validation of the antenna through fabrication and measurement shows good agreement with simulations, establishing that this design provides a superior alternative to other MIMO antennas by combining compactness, flexibility, high gain, enhanced isolation, and compliance with safety standards. It is evident that this work addresses critical challenges in MIMO antenna design, offering a practical solution for high-performance Sub-6 GHz 5G communication systems.

## Figures and Tables

**Table 1:** Single-element antenna parameter dimensions.

Parameter	Ws	Ls	W	L	Lg	a	b	c	d	e	f	g	h	i
Value (in mm)	20	20	0.8	8	6.8	4	7	1	7	5.5	4	1	1	1.2

**Table 2:** The dimensions of the 1 × 2 array antenna.

Parameter	Ws	Ls	g	z1	z2	z3	z4	z5	z6	z7	z8
Value (in mm)	50	26	30	8	29	0.55	6	0.8	10	6.4	12.8

**Table 3:** Comparison between the proposed MIMO configuration and the current literature.

Ref.	Dimension (mm^3^)	Impedance Bandwidth (GHz)	I* (dB)	Flexibility	ECC	DG	MEG	TARC	SAR (W/kg)
[2]	49.09 × 25 × 1.6	3.30–3.85	10	No	~0	~10	0 dB (ratio)	<−10	-
[4]	150 × 75 × 0.8	3.3–4.2 4.4–5 5.15–5.85	14	No	<0.05	-	-	-	-
[13]	160 × 70 × 0.787	5.60–5.67	30	No	<0.5	~10	-	-	0.0989 (10 g) 0.101(1 g)
[23]	103.8 × 68 × 7	3.55–4.2 3.6–4.9 5.15–5.925	10	No	<0.1	-	-	-	-
[24]	150 × 75 × 7	3.3–3.8 4.1–5.8	15	No	<0.03	-	-	<−10	0.179 (1 g)
**Prop**	**57 × 50 × 0.1**	**3.6–3.8** **5.65–5.95**	**22**	**Yes**	**<0.05**	**>9.99**	**−3 dB**	**<−10**	**At 3.75 GHz** **0.0702 (1 g)** **0.25 (10 g)** **At 5.9 GHz** **0.175 (1 g)** **0.507 (10 g)**

I*—isolation.

## Data Availability

The data are provided in the manuscript.
